# Supplementary material for: Cross-sectional survey on adult acute kidney injury in Chinese ICU: the study protocol (CARE-AKI)
Source: BMJ Open. 2018 Jun 6;8(6):e020766. doi: 10.1136/bmjopen-2017-020766 (PMC6009469; doi:10.1136/bmjopen-2017-020766)
Supplement: Author's manuscript [file bmjopen-2017-020766.draft_revisions.pdf]

BMJ Open is committed to open peer review. As part of this commitment we make the peer review history of every article we publish publicly available.

When an article is published we post the peer reviewers' comments and the authors' responses online. We also post the versions of the paper that were used during peer review. These are the versions that the peer review comments apply to.

The versions of the paper that follow are the versions that were submitted during the peer review process. They are not the versions of record or the final published versions. They should not be cited or distributed as the published version of this manuscript.

BMJ Open is an open access journal and the full, final, typeset and author-corrected version of record of the manuscript is available on our site with no access controls, subscription charges or pay-per-view fees (http://bmjopen.bmj.com).

If you have any questions on BMJ Open's open peer review process please email info.bmjopen@bmj.com

BMJ Open

A cross-section survey on adult acute kidney injury in Chinese ICU : the study protocol

Journal:	*BMJ Open*
Manuscript ID	bmjopen-2017-020766
Article Type:	Protocol
Date Submitted by the Author:	22-Nov-2017
Complete List of Authors:	Yang, Zhenyu<br>Wang, Cong<br>Wang, Hongliang<br>Wang, Sicong<br>Liu, Ruijin<br>Wang, Xu<br>Yu, Kaijiang
**Primary Subject Heading**:	Intensive care
Secondary Subject Heading:	Intensive care, Renal medicine
Keywords:	Cross-sectional study, AUDIT, Acute renal failure < NEPHROLOGY

SCHOLARONE™
Manuscripts

A cross-section survey on adult acute kidney injury in Chinese ICU: the study protocol

Zhenyu Yang [1*], Cong Wang [2*], Hongliang Wang [1], Sicong Wang[3], Ruijin Liu [3], Xu Wang [1], Kaijiang Yu [3]

1. Intensive Care Unit, The Second Affiliated Hospital of Harbin Medical University, Harbin, China

2. Intensive Care Unit, The First Affiliated Hospital of Heilongjiang, University of Chinese Medicine, Harbin, China

3. Intensive Care Unit, Cancer Hospital Affiliated to Harbin Medical University, Harbin, China

Zhenyu Yang and Cong Wang contributed equally to this work.

Corresponding author: Dr. Kaijiang Yu,

Intensive Care Unit

Cancer Hospital Affiliated to Harbin Medical University, Harbin, China;

E-mail: icuyukaijiang@sina.com

For peer review only

Abstract

Introduction

Acute kidney injury (AKI) is one of the most serious complications in ICU (intensive care unit) patients, and has become a major problem in both China and overseas. In order to solve this problem, we designed a national survey of AKI in adult ICU, by summarizing the understanding of AKI among Chinese medical staffs, and by analyzing the most likely etiologies, risk factors and mortality of AKI in Chinese ICU. The study is expected to fill in the gap in the epidemiology of adult AKI in Chinese ICU, and to provide a theoretical foundation for developing more scientific and standardized diagnosis and treatment choices for AKI.

Methods

A multicenter cross-section survey, which involved a total of 35 hospitals and 6,147 patients from 23 provinces, 4 municipalities, and 5 autonomous regions, was utilized in this study. All patients who met the inclusion criteria were eligible to apply for the enrollment of the study, which covered both the baseline data and clinical data obtained during diagnosis and treatment.

Ethics and dissemination

Ethical approval was obtained from the ethics committee of Harbin Medical University Cancer Hospital (registration number KY2017-21). The findings of this review will be communicated through peer-reviewed publications and scientific presentations.

For peer review only

Strength of the study

- The largest cross-sectional study of AKI up-to-date among adult ICU patients in China
- Comprehensive research program
- This study was a national (continental) cross-sectional survey, and a total of 23 provinces, 4 municipalities, and 5 autonomous regions were involved
- The aim of this study was to assess the etiology, diagnosis, treatment, and prognosis of AKI in China
- This survey also employed a medical staff questionnaire, which could be used to understand the knowledge and awareness of AKI among Chinese medical staffs

Limitation of the study

- Because of the uneven distribution of medical resources in China, Tibet was not involved in this study and may cause certain information biases
- The difference in the qualifications of doctors and nurses studied by the questionnaire may also cause certain information biases

For peer review only

INTRODUCTION

Acute kidney injury (AKI), one of the most common diseases in critically ill patients, has become a worldwide public health problem due to its high mortality and poor prognosis[1]. At present, due to the differences in the definition of acute renal failure (ARF), the results of relevant epidemiological investigations are quite different. Previous studies have reported an incidence of 5%~20% in critically ill patients. Among them, ARF patients suffering from severe infection accounted for 11%~64%, with a mortality of 10%~50% [2] . About 13 million of hospitalized patients suffer from AKI, which caused about 1.7 million deaths and more than 85% of the mortality occurred in developing countries with a low income. In recent years, the incidence of AKI has further increased in developing countries [3-4] . In 2012, the Kidney Disease: Improving Global Outcomes (KDIGO) guidelines proposed a new definition and staging criteria for AKI. The new definition of AKI has brought tremendous changes in basic research and conventional clinical practice, thus improving the sensitivity of AKI diagnosis and the ability to more accurately predict the prognosis of AKI. In 2013, the International Society of Nephrology (ISN) issued a global appeal for the "0 by 25" program, which was committed to the prevention and treatment of AKI. The goal of the program was that, by 2025, the preventable mortality of AKI would be reduced to zero [5] . This is a great opportunity yet a big challenge for medical professionals in the field of critical care nephrology worldwide, especially in the developing countries. As the largest developing country in the world, China would be able to provide valuable information to improve the knowledge of AKI and to reduce its mortality. Thus, we designed this project with the aim to assess the etiology, diagnosis, treatment, and prognosis of AKI in China, thus providing more data for AKI study around the world. By comparing AKI epidemiology, diagnosis and treatment among different countries in the world, the clinical practice of AKI treatment in China is expected to be improved.

METHODS AND ANALYSIS

Study design and participants

This study was a national (continental) cross-sectional survey, and a total of 23 provinces, 4 municipalities, and 5 autonomous regions were involved. Due to the special population distribution in Beijing, Shanghai and Guangzhou, 2 tertiary first-class hospitals from each of these cities were included in the survey, whereas 1 tertiary first-class hospital from each of other provinces and hospitals was included in the survey. Thus, the total number of hospitals included in the research was 35. The total number of patients included in this study was 6,147, as calculated from the following formula.

Formula for sample size estimation:

Formula for infinite sample population : $$n=\frac{\mu_{\alpha/2}^2 * \pi * (1-\pi)}{\delta^2}$$

$\mu_{\alpha/2}^2=1.96$ π (population rate) =0.02 δ (allowable error) =0.01

$$=1.96^2*0.2*(1-0.2)/0.01^2$$

$$\approx 6147$$

For peer review only - http://bmjopen.bmj.com/site/about/guidelines.xhtml

1. Inclusion criteria

(1) ICU patients receiving consecutive treatments through the 30-day duration of the cross-sectional study;

(2) ⩾ 18 years old;

2. Exclusion criteria

(1) CKD patients;

(2) Patients who underwent renal transplantation;

(3) Patients who underwent routine analysis.

Methods

This study was a national, multicenter, prospective, randomized and cross-sectional clinical survey, which was composed of five parts. The necessary tables of the cross-sectional study were filled out to record related data after patient recruitment (the specific processes are shown in Figure 1). The survey period was from 8: 00 a. m. on the first day to 8: 00 a. m. on the 31st day of the study.

Basic information and daily data of the patients

1. Basic information.

2. The main diagnosis in the ICU and basic creatinine values, etc.

3. APACHE II score, GCS score and SOFA score (the highest score within a 24h period).

The investigation of AKI patients

1. The time of AKI diagnosis, diagnosis criteria and staging;

2. Nephrotoxin drugs used prior to the diagnosis of AKI.

3. The etiology of AKI (pre-renal, renal, post-renal).

4. Life support and its modes.

5. Application of vasoactive drugs and the situation of RRT treatment.

6. The severity and prognosis of AKI.

The general condition of participating ICUs

The general condition of participating ICUs included ICU type, the number of beds in the ICU, patient number, the number and model of blood purification machines, total number of RRT in the survey period, and the annual total number of patients in the ICU.

Survey for ICU doctors

The basic information of participating doctors and their knowledge regarding AKI diagnosis, treatment, and kidney replacement treatment.

For peer review only

Survey for ICU nurses

The basic information of participating nursing staffs and their knowledge about the operation of the blood purification machines.

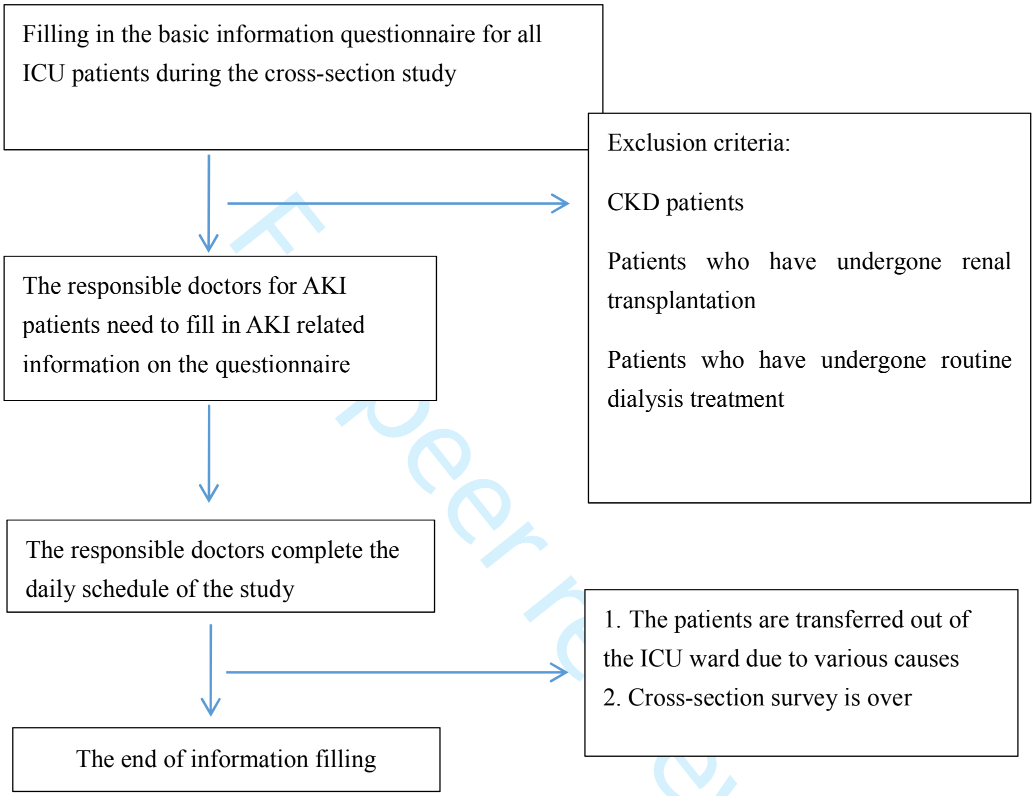

Figure 1 The filling processes of the adult AKI cross-sectional survey

Research protocol

1. This research has been mainly divided into the following sections: the comparison of etiology, incidence, mortality, ICU cost, and total hospital stay among AKI patients, and the relationship between the severity and prognosis of AKI.

2. Factors related to prognosis among AKI patients (application of diuretics; infusion of blood products, infusion of crystalloid or colloid; intra-abdominal pressure).

3. At the same time, the prognosis and mortality, hospital spending, duration of ICU stay and total hospitalization time for AKI patients with sepsis were collected and compared with other etiological factors, so as to study the relationship between the severity and prognosis of AKI.

4. The doctor composition, proportion of nursing staffs in ICU, their awareness of AKI, their awareness and implementation of AKI guidelines, and the specific clinical operations were surveyed.

5. Current application of RRT treatment among AKI patients in China. In addition, a further

For peer review only - http://bmjopen.bmj.com/site/about/guidelines.xhtml

research on continuous blood purification was carried out on this basis.

Through the entire research: to study and estimate the incidence and mortality of adult patients in Chinese ICU, and to find the easiest cause of AKI in China; to investigate current practice of AKI diagnosis and treatment in Chinese ICU; to study the effect of kidney replacement on the development and prognosis of AKI; to understand the awareness of AKI among Chinese medical staffs; to fill in the gap regarding the knowledge of AKI research in developing countries; and to provide more data for worldwide research on the diagnosis and treatment of AKI.

Study ethics and the protection of patient rights

This study was approved by the ethics committee of the participating hospitals. All patients volunteered to participate, and signed the informed consent. All patient information was kept confidential.

DISCUSSION

Kidney is an important excretory and endocrine organ in the body, and appropriate kidney functions are important to maintain body homeostasis and to ensure the normal metabolism in the body. In ICU, due to the underlying diseases, the onset of severe infection, multiple trauma, and a variety of shock symptoms, it is highly likely to induce AKI or to exacerbate the pre-existing condition of AKI. Kidney often interacts with other important organs (such as heart, lung, liver, brain), and hence deteriorated kidney functions can further aggravate the patients' conditions and affect the prognosis of the patients. Therefore, the maintenance of renal function is often critical. However, due to the differences among different countries and regions, their incidence of AKI was significantly different [6]. In addition, there is a lack of uniform standards in terms of early diagnosis of AKI, its standardized treatment strategies, and the application of renal replacement therapy (RRT). In recent years, the incidence of AKI in developing countries has increased [7]. Most recent studies have focused on the status of AKI in developed countries, whereas the reports on AKI in developing countries are scarce. Due to their regional differences and inequity, it is not easy to conduct an epidemiological AKI survey in developing countries. Many articles have pointed out the lack of reliable data to derive the incidence of AKI in Africa, Asia, and Latin America [8-10]. Secondly, the definitions of AKI in different parts of the world are quite different. The epidemiological data in developing countries are often derived from single-center studies in better developed regions of these countries, thus leading to the underestimation regarding the incidence of AKI in these countries, although the overall incidence of AKI has been increasing year by year[11-12]. For example, a study in Saudi Arabia in 2003 showed that the incidence of AKI in hospitalized patients was 2.3/1000 [13-14], whereas in 2007, a medical center in India reported an AKI incidence of 6.6/1000 among hospitalized patients [15]. Nevertheless, in Southeast Asia, due to the monsoon climate, malaria, leptospirosis and gastrointestinal tract infection, the incidence of AKI was up to 18%~ 24% [16]. Data depicting the epidemiological characteristics of AKI in China, the largest developing country in the world, are more interesting. A national and multicenter AKI epidemiology study, which was conducted in China and published in 2015, focused primarily on hospitalized patients, and showed that the incidence of AKI, the mortality and the misdiagnosis rate among their subjects were 1%～2%, 12.4% and 74.2%, respectively [17]. Although the above study showed the basic and

epidemiological characteristics of AKI among hospitalized patients in China, little evidences are currently available regarding the epidemiological characteristics of AKI patients in Chinese ICU. Therefore, the current situation of AKI diagnosis and treatment in Chinese ICU remains unclear. This study will answer these questions and is expected to provide important information for future treatment of AKI patients in Chinese ICU.

This study mainly covered several important aspects: diagnosis, etiology, life support and monitoring, prognostic indicators, and medical staffs' awareness of AKI.

Diagnosis criteria and the etiology of AKI

The differences in AKI diagnostic criteria can lead to different time of diagnosis and hence affect subsequent treatments. We first investigated the time and criteria of AKI diagnosis among patients in ICU. The following diagnostic criteria were included: RIFLE in 2005, the AKIN criteria in 2007, and the KDIGO criteria in 2012. The KDIGO standard will also be used for AKI staging. At the same time, according to the KDIGO guidelines, the etiology of AKI, especially the relevant exposure factors such as the history of systemic infection, severe disease, shock, burn, trauma, cardiac surgery (especially surgeries involving extracorporeal circulation) and large non-cardiac surgery, the application of nephrotoxic drugs, the application of contrast agents, and the exposure to poisonous plants or animals, [18] will be investigated.

Life support and monitoring

In this cross-sectional study, we first investigated whether the AKI patients in ICU have received mechanical ventilation, and the mode of such mechanical ventilation was recorded. The abdominal pressure in AKI patients was monitored during the first 24 hours after admission, with the highest and lowest values recorded, so as to assess the effect of abdominal pressure on the incidence, severity and prognosis of AKI. Secondly, it has been reported that about 59~70% of patients suffering from renal injury have used diuretics prior to urologist consultation or at the beginning of the RRT [19]. An earlier study has shown that loop diuretics could improve renal functions, while furosemide could remove the particles blocking the renal tubules and increased renal blood flow [20]. However, many studies failed to show the beneficial effect of such drugs on renal functions, and some studies even showed that the application of such drugs increased the mortality [21]. In 2012, the KIDGO guidelines no longer recommended routine use of diuretics. Thus, in our survey, the information on the use of diuretics, such as diuretic types, dose, drug delivery methods and related hemodynamic indexes, was monitored and recorded.

The debate about the timing of RRT treatment in AKI patients has intensified in recent years, although no conclusion has been reached yet [22]. In this study, the main reasons for RRT treatment, the initiation time of RRT treatment and its outcomes were recorded.

A wide range of literatures has studied the effect of different vasoactive drugs on renal functions, on the improvement of renal blood flow and on the duration of RRT treatment, although their results have been inconsistent [23-24]. In this study, we monitored the use of vasoactive drugs, including dopamine, norepinephrine, epinephrine and dobutamine, in AKI patients in ICU.

Renal hypoperfusion is one of the major causes to trigger AKI. Although aggressive fluid resuscitation is required, there has been controversy regarding the type of fluid to be used in

For peer review only - http://bmjopen.bmj.com/site/about/guidelines.xhtml

resuscitation[25-26]. More evidences have suggested that hydroxyethyl starch increases the mortality in patients with severe AKI, and increases the incidence and severity of AKI. On the other hand, in critically ill patients, hypertonic chloride liquid is used intravenously during fluid resuscitation, which may induce or aggravate hyperchloremia and metabolic acidosis, eventually leading to renal vasoconstriction and reduced glomerular filtration rate. In this study, we also collected the data related to fluid resuscitation, including the fluid type as well as the intake and output volume in 24 hours.

Prognostic indicators

A previous large-scale meta-analysis analyzed 312 studies which were conducted from 2004 to 2012 and involved 49,147,878 patients, and re-graded all these 312 studies according to the KDIGO standard. The results showed that the general incidence of AKI was as high as 23.2% worldwide, with 11.5% of AKI in stage 1, 4.8% in stage 2 and 4% in stage 3, respectively. The total mortality in AKI patients was about 23%, and the mortality of AKI in stage 1, stage 2 and stage 3 was 15.9%, 28.5% and 47.8%, respectively [27]. The mortality among AKI patients from different countries was affected by of the status of medical treatment, the level of economic development and the geographical location. A study in 2015 compared the mortality in AKI patients among different ICUs in different countries. As a result, it was found that the mortality of AKI patients in different ICU was quite different. However, if the different levels of income, different medical expenses in different countries, and the GDP and latitude of these countries were considered in the statistical analysis, the mortality in different groups of patients was similar [28]. In this study, we also focused on the prognosis, staging (KDIGO) and clinical outcomes of AKI patients. The clinical outcomes included discharge mortality, 28-day mortality, and the need for long-term renal replacement therapy after discharge.

Medical staff questionnaire

In the third part of this cross-sectional survey, the basic data of participation units were collected. The doctors and nurses in the ICU would be evaluated using questionnaires regarding their knowledge and training for serving AKI patients. In addition, the composition of the doctors and the nursing staff ratio index were also recorded.

The innovative aspects of this survey are as follows: (1) This is the first time to conduct a national survey on the epidemiology of AKI among ICU patients in China. At least one representative hospital has been recruited from each province/municipality or autonomous region. Thus, the data would reflect the real status of AKI in entire China. (2) This survey also uses a medical staff questionnaire, which can be used to understand the knowledge and awareness of AKI among Chinese medical staffs. Through this survey, we expect to obtain the data that can help us to improve our clinical practice in the treatment of AKI.

For peer review only

COMPETING INTERESTS STATEMENT

The authors have declared no competing interests regarding their effort spent in the current study.

CONTRIBUTORSHIP STATEMENT

No person other than the authors listed here has contributed significantly to the manuscript preparation.

All persons listed here as authors contributed to the preparation of the manuscript and the International Committee of Medical Journal Editors (ICMJE)'s criteria for authorship were met. Specifically, the following contributions were made by respective authors:

Kaijiang Y:

1. Acted as the project's principal investigator and made substantial contributions to the conception and design of the study;

2. Agreed to be responsible for all aspects of the study;

3. Approved the final version of the draft before its publication.

Zhenyu Y, Cong W:

1. Collected and studied a large number of relevant references;

2. Drafted the study report and revised it;

3. Approved the final version of the draft before its publication.

Hongliang W , Sicong W , Ruijin L， Xu W:

1. Collected important background information related to the study;

2. Contributed substantially to the acquisition, analysis, and interpretation of data;

3. Drafted the study report and revised it;

4. Approved the final version of the draft before its publication.

For peer review only

STUDY STATUS

All preliminary work has finished. The data collection of this cross-section survey will be carried out in January and February, 2018.

FUNDING STATEMENT

None.

DATA SHARING STATEMENT

No additional data are available.

For peer review only

REFERENCES

［1］Susantitaphong P, Cruz DN, Cerda J, et al. World incidence of AKI:a meta-analysis. Clin J Am Soc Nephrol, 2013, 8(9): 1482-93.

［2］Barrantes F, Tian J, Vazquez R, et al. Acute kidney injury criteria predict outcomes of critically ill patients［J］. Crit Care Med, 2008,36(5): 1397-403.

［3］Lewington AJ, Cerda J, Mehta RL. Raising awareness of acute kidney injury: a global perspective of a silent killer［J］. Kidney Int,2013, 84(3): 457-67.

［4］Mehta RL, Cerdá J, Burdmann EA, et al. International society of nephrology's 0by25 initiative for acute kidney injury (zero preventable deaths by 2025): a human rights case for nephrology［J］. Lancet, 2015, 385(9987): 2616-43.

［5］Remuzzi G, Horton R. Acute renal failure: an unacceptable death sentence globally[J]. Lancet, 2013, 382(9910): 2041-2.

［6］Li PK, Burdmann EA, Mehta RL,et al.Acute kidney injury: Global health alert. J Nephropathol, 2013 , 2(2):90-7.

［7］Susantitaphong P ,Cruz DN, Cerda J, et al. World incidence of AKI : a meta – analysis. Clin J AM Soc Nephrol, 2013,8(9):1482-1493

［8］Naicker S，Aboud O，Gharbi MB． Epidemiology of acute kidney injury in Africa．Semin Nephrol，2008, 28(4):348 －353．

［9］Jha V，Chugh KS． Community － acquired acute kidney injury in Asia． Semin Nephrol，2008，28(4):330 －347．

［10］Lombardi Ｒ，Yu L，Younes － Ibrahim M，et al． Epidemiology of acute kidney injury in Latin America． Semin Nephrol，2008，28 (4):320 －329．．

［11］Cruz DN, Ricci Z, Ronco C .Clinical review :RIFLE and AKIN-time for reappraisal .Crit Care ,2009,13(3) :211.

［12］Wald R, McArthur E, Adhikari NK, et al .Changing incidence and outcomes following dialysis-requiring acute kidney injury among critically ill adults :a population-based cohort study .AM J Kidney Dis,2015,65(6):870-877.

［13］Al－Homrany M．Epidemiology of acute renal failure in hospitalized patients: experience from southern Saudi Arabia. East Mediterr Health J，2003， 9(5－6):1061－1067．

［14］Kohli HS，Bhat A，Jairam A，et al． Predictors of mortality in acute renal failure in a developing country:a prospective study． Ｒen Fail，2007， 29(4):463 －469．

［15］Wang Y，Cui Z，Fan M． Ｒetrospective analysis on Chinese patients diagnosed with acute renal failure hospitalized during the last decade (1994－2003)．Am J Nephrol，2005，25(5):514－519．

For peer review only

［16］Chitalia VC，Almeida AF，Rai H，et al．Is peritoneal dialysis adequate for hypercatabolic acute renal failure in developing countries? Kidney Int，2002, 61(2):747 －757．

［17］Yang L, Xing G, Wang L, Wu Y,et al.Acute kidney injury in China: a cross-sectional survey. Lancet, 2015,386(10002):1465-71.

［18］KDIGO. Clinical practice guideline for acute kidney injury [J]. Kidney Inter, 2012 ,2 :1-138.

［19］Mehta RL, Pascual MT, Soroko S, et al.Diuretics, mortality, and nonrecovery of renal function in acute renal failure. JAMA,2002,288 (20):2547-53.

［20］Ludens JH, Williamson HE.Effect of furosemide on renal blood flow in the conscious dog.Proc Soc Exp Biol Med, 1970, 133(2):513-5.

［21］Uchino S, Doig GS, Bellomo R, et al. Diuretics and mortality in acute renal failure.Crit Care Med, 2004,32(8):1669-77.

［22］Karvellas CJ, Farhat MR, Sajjad I, et al. A comparison of early versus late initiation of renal replacement therapy in critically ill patients with acute kidney injury: a systematic review and meta-analysis. Crit Care, 2011,15(1):R72.

［23］De Backer D, Biston P, Devriendt J, et al.Comparison of dopamine and norepinephrine in the treatment of shock. N Engl J Med, 2010 ,362(9):779-89.

［24］Lauzier F, Levy B, Lamarre P, et al.Vasopressin or norepinephrine in early hyperdynamic septic shock: a randomized clinical trial.Intensive Care Med, 2006 ,32(11):1782-9.

［25］Annane D, Siami S, Jaber S, et al. Effects of fluid resuscitation with colloids vs crystalloids on mortality in critically ill patients presenting with hypovolemic shock: the CRISTAL randomized trial. JAMA., 2013 ,310(17):1809-17.

［26］Myburgh JA, Finfer S, Bellomo R, et al. Hydroxyethyl starch or saline for fluid resuscitation in intensive care. N Engl J Med, 2012 , 367(20):1901-11.

［27］Krajewski ML ,Raghunathan K，Paluszkiewicz SM，et al. Meta-analysis of high- versus low-chloride content in perioperative and critical care fluid resuscitation. Br J Surg,2015, 102(1):24-36.

［28］Hoste EA, Bagshaw SM, Bellomo R ,et al. Epidemiology of acute kidney injury in critically ill patients : the multinational AKI-EPI study. Intensive Care Med,2015,41(8):1411-1423.

For peer review only

BMJ Open

A cross-sectional survey on adult acute kidney injury in Chinese ICU : the study protocol (CARE-AKI)

Journal:	*BMJ Open*
Manuscript ID	bmjopen-2017-020766.R1
Article Type:	Protocol
Date Submitted by the Author:	18-Feb-2018
Complete List of Authors:	Yang, Zhenyu<br>Wang, Cong<br>Wang, Hongliang<br>Wang, Sicong<br>Liu, Ruijin<br>Wang, Xu<br>Yu, Kaijiang
**Primary Subject Heading**:	Intensive care
Secondary Subject Heading:	Intensive care, Renal medicine
Keywords:	Cross-sectional study, AUDIT, Acute renal failure < NEPHROLOGY

SCHOLARONE™ Manuscripts

For peer review only

A cross-sectional survey on adult acute kidney injury in Chinese ICU: the study protocol (CARE-AKI)

Zhenyu Yang [1*], Cong Wang [2*], Hongliang Wang [1], Sicong Wang[3], Ruijin Liu [3], Xu Wang [1], Kaijiang Yu [3]

1. Intensive Care Unit, The Second Affiliated Hospital of Harbin Medical University, Harbin, China

2. Intensive Care Unit, The First Affiliated Hospital of Heilongjiang, University of Chinese Medicine, Harbin, China

3. Intensive Care Unit, Cancer Hospital Affiliated to Harbin Medical University, Harbin, China

Zhenyu Yang and Cong Wang contributed equally to this work.

Corresponding author: Dr. Kaijiang Yu,

Intensive Care Unit

Cancer Hospital Affiliated to Harbin Medical University, Harbin, China;

E-mail: icuyukaijiang@sina.com

For peer review only

Abstract

Introduction

Acute kidney injury (AKI) is one of the most serious syndromes for ICU (intensive care unit) patients, and is being a mysterious problem in clinical practice worldwide. Due to unknown etiology and mechanism, there are varied awareness for AKI diagnosis and treatment in China, resulting in underestimated incidence and poor prognosis. In order to solve this problem, we design this national survey of AKI in adult ICU. Various indexes are included and analyzed to classify epidemiology of adult AKI in Chinese ICU, including AKI etiology, risk factors, mortality, prognosis, therapeutic strategies and cognition of ICU medical staffs..

Methods

A multicenter cross-sectional survey, which will involve about 35 hospitals and 6,147 patients from 23 provinces, 4 municipalities, and 5 autonomous regions, is planned in this study. All patients who meet the inclusion criteria are eligible to apply for the enrollment of the study, which covere the baseline demography, clinical performance and follow-up related todiagnosis and treatment.

Conculsion

The study is expected to fill in the gap between China and developed countries, and to provide a theoretical foundation for developing more scientific and standardized approaches for AKI diagnosis and treatment.

Ethics and dissemination

Ethical approval was obtained from the ethics committee of Harbin Medical University Cancer Hospital (registration number KY2017-21). The findings of this review will be communicated through peer-reviewed publications and scientific presentations.

For peer review only

Strength of the study

- The largest cross-sectional study of AKI up-to-date among adult ICU patients in China
- Comprehensive research program
- This study is national , including about 23 provinces, 4 municipalities, and 5 autonomous regions
- The aim of this study is to assess the etiology, diagnosis, treatment, and prognosis of AKI in China
- This survey also is planned to introduce the medical staff questionnaire, which could help to understand the knowledge and awareness of AKI among Chinese medical staffs

Limitation of the study

- Due to the uneven distribution of economic and medical resources in China, our survey might have the information biases

For peer review only

INTRODUCTION

Acute kidney injury (AKI), one of the most common syndromes in critically ill patients, has become a health problem worldwide due to its high mortality and poor prognosis[1]. Due to current different definition and unknow mechanism of acute renal failure (ARF), the findings of epidemiological investigations are quite different. Previous studies reported an incidence of 5%~20% in critically ill patients[2]. About 13 million of hospitalized patients suffer from AKI, which caused about 1.7 million deaths and more than 85% of the mortality occurred in developing countries with a low income. In recent years, the AKI incidence is increasing in developing countries[3 4]. A national AKI epidemiology study showed the AKI incidence, mortality and misdiagnosis rate were 1%～2%, 12.4% and 74.2% among hospitalized patients in China[5]. In 2012, the Kidney Disease: Improving Global Outcomes (KDIGO) guidelines proposed a new definition and staging criteria for AKI. The new definition of AKI has brought tremendous changes in basic research and conventional clinical practice, thus improving the sensitivity of AKI diagnosis and the ability to more accurately predict the prognosis of AKI. Due to varied medical resource and awareness of AKI, the current situation of AKI diagnosis and treatment remains unclear especially in Chinese multicenter ICU. In 2013, the International Society of Nephrology (ISN) issued a global appeal for the "0 by 25" program, which was committed to the prevention and treatment of AKI. The goal of the program was that, by 2025, the preventable mortality of AKI would be reduced to zero[6]. As the largest developing country in the world, China has the large patient population and different medical background of hospitals. In order to improve the AKI awareness and its prognosis, this study protocol is designed to assess the etiology, diagnosis, treatment, and prognosis of AKI in China ICU. Therefore, the results will be expected to provide important information for future management of AKI patients in Chinese.

METHODS AND ANALYSIS

Study design

This study is a national cross-sectional survey, involving about 23 provinces, 4 municipalities, and 5 autonomous regions. One tertiary first-class hospital from each provinces is included at least. Due to the large population and advanced medical resources, two tertiary first-class hospitals are included in Beijing, Shanghai and Guangzhou. As planned, the total number of hospitals will be 35. The total number of patients included in this study is estimated to reach 6,147, as calculated from the following formula.

Formula for sample size estimation:

Formula for infinite sample population : $n=\frac{\mu_{\alpha/2}^2 * \pi * (1-\pi)}{\delta^2}$

$\mu_{\alpha/2}^2=1.96$ π (population rate) =0.02 δ (allowable error) =0.01

$=1.96^2*0.2*(1-0.2)/0.01^2$

≈ 6147

Sampling criteria

For peer review only

Inclusion criteria

(1) ICU patients receiving consecutive treatments through the cross-sectional period;

(2) ⩾ 18 years old;

Exclusion criteria

(1) CKD patients;

(2) Patients who underwent renal transplantation;

(3) Patients who underwent routine analysis.

Date collection

This study is a national, multicenter, prospective, randomized and cross-sectional clinical survey, including five aspects. The standardized forms are created to record related data after patient recruitment (the specific processes are shown in Figure 1). Through the consensus discussion, the study committee will be elected and supervise the study procedure. The survey period is intended to be from 8: 00 a. m. on the first day to 8: 00 a. m. on the 31st day of the study.

a. Patient demography and clinical performance

1. Basic information.

2. The main diagnosis in the ICU and basic creatinine values, etc.

3. APACHE II score, GCS score and SOFA score (the highest score within a 24h period).

b. The investigation of AKI patients

1. The time of AKI diagnosis, diagnosis criteria and staging;

2. Nephrotoxin drugs used prior to the diagnosis of AKI.

3. The etiology of AKI (pre-renal, renal, post-renal).

4. Life support and its modes.

5. Application of vasoactive drugs and the prescription of RRT treatment.

6. The severity and prognosis of AKI.

c. ICU facility and infrastructure

The general condition of participating ICUs are recorded, including ICU type, the bed number, patient population, blood purification machines, RRT strategy and the annual cases in the ICU.

d. Survey for ICU doctors

The participating doctors are inquired for their personal information, clinical experience, and especially their knowledge/awareness about AKI, including diagnosis, treatment, and kidney replacement treatment.

For peer review only

e. Survey for ICU nurses

The participating nursing staff are inquired for their personal information, clinical experience, and especially their knowledge/awareness about AKI and blood purification machines.

Measurements and statistical analysis

1. The results are mainly compared for these index: AKI etiology, incidence, mortality, ICU cost, total hospitalization and prognosis.

2. Risk factors are screened to evaluate AKI prognosis, including diuretics, blood infusion, infusion of crystalloid or colloid, intra-abdominal pressure etc.

3. AKI patients with sepsis are filtered to apply the subgroup analysis among AKI cases, inorder to classify to the relationship between the severity and prognosis of AKI.

4. ICU facility, team composition, medical awareness of staffs and AKI guideline implementations are surveyed to illuminate the district variance due to different medical resources.

5. Current application and prescription of RRT treatments are counted among AKI patients and evaluated for their efficiency in China, in order to foster the standardized clinical protocol. The further research on continuous blood purification will be carried out on this basis.

Study ethics and the protection of patient rights

This study was approved by the ethics committee of the participating hospitals. All participating patients are informed and sign the written consent. All patient information are kept confidential.

DISCUSSION

Kidney is an important excretory and endocrine organ in the body, which is critical to maintain body homeostasis and normal metabolism. In ICU, due to the underlying diseases, such as severe infection, multiple trauma, and a variety of shock symptoms, it is highly likely to induce AKI or exacerbate the pre-existing condition of AKI. Kidney dysfunctions can further aggravate the patients' conditions and affect their prognosis. Prevent, diagnosis and treatment of AKI are often critical for public health worldwide. The AKI etiology and mechanism are still unknown. There is also no consensus standards in terms of AKI early diagnosis, treatment strategy, and renal replacement therapy (RRT).Due to the different definition among countries, AKI incidence was reported to be significantly varied[7]. Most recent studies were from developed countries, whereas the reports are scarce in developing countries. But the AKI incidence is increasing and underestimated in developing countries[7-9]. Many researches pointed out the epidemiological data was also not reliable for those single-center studies in Africa, Asia, and Latin America[10-12]. For example, a study in Saudi Arabia in 2003 showed that the AKI incidence was 2.3/1000 of hospitalized patients[13 14], while a single center in India reported the AKI incidence of 6.6/1000 among hospitalized patients in 2007[15]. In Southeast Asia, due to the monsoon climate, malaria, leptospirosis and gastrointestinal tract infection, the AKI incidence was up to 18%~ 24%[16]. A national and multicenter AKI epidemiology study was conducted in China in 2015, which indicated the AKI incidence, mortality and misdiagnosis rate were 1%～2%, 12.4% and 74.2% among hospitalized patients[5]. But the current

For peer review only

situation of AKI diagnosis and treatment in Chinese ICU remains unclear. This survey study mainly cover several critical aspects for managing AKI : diagnosis, etiology, life support and monitoring, prognostic indicators, and medical staffs' awareness. Through our findings, we might will answer these questions and is expected to provide effective and standardized approaches for future AKI management in Chinese ICU.

Diagnosis criteria and the etiology of AKI

The distinct criteria for early AKI results in different time of accurate diagnosis and greatly affect subsequent clinical intervention. The time and criteria of AKI diagnosis are investigated among multicenter ICU, including RIFLE in 2005, the AKIN in 2007, and the KDIGO in 2012. Their sensitivity, specificity and clinical effect are compared to optimize clinical practice of AKI diagnosis. Referring to the KDIGO guidelines, the etiology of AKI is recorded and compared as required, such as the infection history, severe disease, shock, burn, trauma, cardiac surgery (especially surgeries involving extracorporeal circulation) and large non-cardiac surgery, nephrotoxic drugs, contrast agents, and the exposure to poisonous plants or animals[17] . These will illuminate general AKI etiology and region difference, which is important for medical awareness and early prevention of AKI.

ICU Life support concerned with AKI

There are lots of controversial findings about life-support approaches to induce or aggravate AKI in ICU. For this cross-sectional study, we intend to collect more big data to figure out their effect on AKI, especially for mechanical ventilation, diuretics, vasoactive drugs, renal perfusion and RRT,. To assess the effect of abdominal pressure on AKI, ICU patients with mechanical ventilation are enrolled. The mode of mechanical ventilation is recorded. The abdominal pressure in AKI patients should be monitored during the first 24 hours after admission. In 2012, the KIDGO guidelines no longer recommended routine use of diuretics. Loop diuretics were previously reported to improve renal functions, removing the block particles and increasing renal blood flow[18 19]. Further studies failed to verify the beneficial effect, but showed that such drugs increased the mortality[20]. In our survey, the diuretics prescription is recorded in detail to further clear the confusion and correct the medicine knowledge, such as diuretic types, dosage, drug delivery and hemodynamic indexes. Some investigation is applied to study the inconsistent effect of vasoactive drugs on renal function[21 22]. Different drugs are compared in AKI cases, including dopamine, norepinephrine, epinephrine and dobutamine.

Renal perfusion and resuscitation resulted in different outcomes by varied fluid type[23 24]. Hydroxyethyl starch might aggravate the incidence and severity of AKI, even increase the mortality in severe AKI cases. In critically ill patients, hypertonic chloride liquid might induce or aggravate hyperchloremia and metabolic acidosis, and eventually reduce glomerular filtration rate by renal vasoconstriction and. The fluid resuscitation with the fluid type is required to collect, including the intake/output volume in 24 hours. RRT treatment is critical for treating AKI patients, but its prescription and application guideline are with no consensus[25]. Through our large sample multicenter study, we record the different strategy and outcome of RRT treatment to foster standardized clinical approaches.

Prognostic indicators

The AKI mortality from different countries is affected by the medical status, the economic level and the geographical location[26]. If the different social and economic factors of these countries were considered in the statistical analysis, the mortality in different groups was similar[27]. In this study, we focus on the prognosis, staging (KDIGO) and clinical outcomes of AKI patients, such as in-hospital mortality, 28-day mortality, and long-term renal replacement therapy. The ICU facility, local economy and personal characteristics are also introduced to adjust our statistical calculation. More importantly, the difference for AKI prognosis will foster the individual health policy and treatment strategy in China.

Medical staff awareness

The AKI knowledge and awareness are critical for clinical practice. In this cross-sectional survey, the questionnaires are introduced to objectively evaluate medical acquisition of medical staff. The team composition of doctors and the nurse ratio are also recorded. Through our data mining, the results can inspire the further education and team optimization of ICU staffs. The varied finding for doctors' knowledge will be calculated to solve the analysis bias and evaluate the accurate AKI epidemiology in China.

This is the first national survey on the epidemiology of AKI among ICUs in China. As planned, one representative hospital is recruited from each province/municipality or autonomous region. The findings would reflect the real status of AKI in entire China, help to establish standardized guidelines and promote the individual optimization of ICU AKI treatment. This survey also introduces the medical questionnaire for large staff population, which can be used to understand the knowledge and awareness of AKI. Through this survey, we expect to improve our clinical practice in the management of AKI in China. These data would also inspire the further studies in other developing countries.

COMPETING INTERESTS STATEMENT

The authors have declared no competing interests regarding their effort spent in the current study.

CONTRIBUTORSHIP STATEMENT

No person other than the authors listed here has contributed significantly to the manuscript preparation.

All persons listed here as authors contributed to the preparation of the manuscript and the International Committee of Medical Journal Editors (ICMJE)'s criteria for authorship were met. Specifically, the following contributions were made by respective authors:

Kaijiang Y:

1. Act as the project's principal investigator, and contribute to the study conception and design;

2. Agree to be responsible for all aspects of the study;

3. Approve the final draft before its publication.

For peer review only

Zhenyu Y, Cong W:

1. Collect and study a large number of relevant references;

2. Draft the study report and revise it;

3. Approve the final draft before its publication.

Hongliang W , Sicong W , Ruijin L， Xu W:

1. Collecte important background information related to the study;

2. Contribute substantially to the acquisition, analysis, and interpretation of data;

3. Draft the study report and revise it;

4. Approve the final draft before its publication.

For peer review only

STUDY STATUS

All preliminary work has finished. The data collection of this cross-section survey will be carried out in 2018.

FUNDING STATEMENT

This project is funded by National Natural Science Foundation of China (No. 81571871 and 81770276).

DATA SHARING STATEMENT

No additional data are available.

REFERENCES

1. Susantitaphong P, Cruz DN, Cerda J, et al. World Incidence of AKI: A Meta-Analysis. *Clinical Journal of the American Society of Nephrology* 2013;8(9):1482-93.
2. Barrantes F, Tian J, Vazquez R, et al. Acute kidney injury criteria predict outcomes of critically ill patients. *Crit Care Med* 2008;36(5):1397-403.
3. Lewington AJP, Cerdá J, Mehta RL. Raising awareness of acute kidney injury: a global perspective of a silent killer. *Kidney International* 2013;84(3):457-67.
4. Mehta RL, Cerdá J, Burdmann EA, et al. International Society of Nephrology's 0by25 initiative for acute kidney injury (zero preventable deaths by 2025): a human rights case for nephrology. *The Lancet*;385(9987):2616-43.
5. Yang L, Xing G, Wang L, et al. Acute kidney injury in China: a cross-sectional survey. *The Lancet* 2015;386(10002):1465-71.
6. Remuzzi G, Horton R. Acute renal failure: an unacceptable death sentence globally. *The Lancet*;382(9910):2041-42. doi: 10.1016/S0140-6736(13)62193-5
7. Kam Tao Li P, Burdmann EA, Mehta RL, et al. Acute kidney injury: Global health alert. *J Nephropathol* 2013;2(2):90-7. doi: 10.12860/JNP.2013.15
8. Cruz DN, Ricci Z, Ronco C. Clinical review: RIFLE and AKIN – time for reappraisal. *Critical Care* 2009;13(3):211. doi: 10.1186/cc7759
9. Wald R, McArthur E, Adhikari NKJ, et al. Changing Incidence and Outcomes Following Dialysis-Requiring Acute Kidney Injury Among Critically Ill Adults: A Population-Based Cohort Study. *American Journal of Kidney Diseases* 2015;65(6):870-77.
10. Naicker S, Aboud O, Gharbi MB. Epidemiology of Acute Kidney Injury in Africa. *Seminars in Nephrology* 2008;28(4):348-53.
11. Jha V, Chugh KS. Community-Acquired Acute Kidney Injury in Asia. *Seminars in Nephrology* 2008;28(4):330-47.
12. Lombardi R, Yu L, Younes-Ibrahim M, et al. Epidemiology of Acute Kidney Injury in Latin America. *Seminars in Nephrology* 2008;28(4):320-29.
13. Al-Homrany M. Epidemiology of acute renal failure in hospitalized patients: experience from southern Saudi Arabia. *East Mediterr Health J* 2003;9(5-6):1061-7.
14. Kohli HS, Bhat A, Jairam A, et al. Predictors of Mortality in Acute Renal Failure in a Developing Country: A Prospective Study. *Renal Failure* 2007;29(4):463-69.
15. Wang Y, Cui Z, Fan M. Retrospective Analysis on Chinese Patients Diagnosed with Acute Renal Failure Hospitalized during the Last Decade (1994–2003). *American Journal of Nephrology* 2005;25(5):514-19.
16. Chitalia VC, Almeida AF, Rai H, et al. Is peritoneal dialysis adequate for hypercatabolic acute renal failure in developing countries? *Kidney International* 2002;61(2):747-57.
17. KDIGO. Clinical practice guideline for acute kidney injury. *Kidney International* 2012;2(1):1-138.
18. Mehta RL, Pascual MT, Soroko S, et al. Diuretics, mortality, and nonrecovery of renal function in acute renal failure.

For peer review only

JAMA 2002;288(20):2547-53.

19. Ludens JH, Williamson HE. Effect of furosemide on renal blood flow in the conscious dog. *Proc Soc Exp Biol Med* 1970;133(2):513-5.
20. Uchino S, Doig GS, Bellomo R, et al. Diuretics and mortality in acute renal failure*. *Critical Care Medicine* 2004;32(8):1669-77. f
21. De Backer D, Biston P, Devriendt J, et al. Comparison of Dopamine and Norepinephrine in the Treatment of Shock. *New England Journal of Medicine* 2010;362(9):779-89.
22. Lauzier F, Lévy B, Lamarre P, et al. Vasopressin or norepinephrine in early hyperdynamic septic shock: a randomized clinical trial. *Intensive Care Medicine* 2006;32(11):1782-89.
23. Annane D, Siami S, Jaber S, et al. Effects of fluid resuscitation with colloids vs crystalloids on mortality in critically ill patients presenting with hypovolemic shock: The cristal randomized trial. *JAMA* 2013;310(17):1809-17.
24. Myburgh JA, Finfer S, Bellomo R, et al. Hydroxyethyl Starch or Saline for Fluid Resuscitation in Intensive Care. *New England Journal of Medicine* 2012;367(20):1901-11.
25. Karvellas CJ, Farhat MR, Sajjad I, et al. A comparison of early versus late initiation of renal replacement therapy in critically ill patients with acute kidney injury: a systematic review and meta-analysis. *Critical Care* 2011;15(1):R72.
26. Krajewski ML, Raghunathan K, Paluszkiewicz SM, et al. Meta-analysis of high- versus low-chloride content in perioperative and critical care fluid resuscitation. *British Journal of Surgery* 2015;102(1):24-36.
27. Hoste EAJ, Bagshaw SM, Bellomo R, et al. Epidemiology of acute kidney injury in critically ill patients: the multinational AKI-EPI study. *Intensive Care Medicine* 2015;41(8):1411-23.

For peer review only

Figure Legend

Figure 1: This study is a national, multicenter, prospective, randomized and cross-sectional clinical survey in China . Thus, a high-quality cross-sectional survey is needed and the process of performing this study can be found in figure 1, which will be separated into four parts:

For peer review only

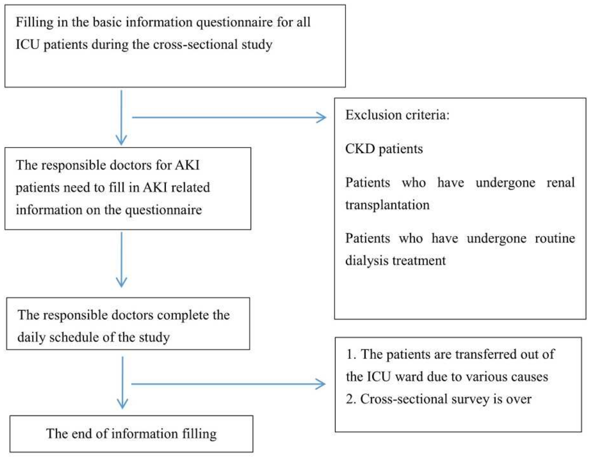

Figure 1 The filling processes of the adult AKI cross-sectional survey

296x419mm (300 x 300 DPI)
